# An Overview of Recycling Wastes into Graphene Derivatives Using Microwave Synthesis; Trends and Prospects

**DOI:** 10.3390/ma16103726

**Published:** 2023-05-14

**Authors:** Nuralmeera Balqis, Badrul Mohamed Jan, Hendrik Simon Cornelis Metselaar, Akhmal Sidek, George Kenanakis, Rabia Ikram

**Affiliations:** 1 Department of Chemical Engineering, Faculty of Engineering, Universiti Malaya, Kuala Lumpur 50603, Malaysia; nuralmeerabalqis@gmail.com; 2 Centre of Advanced Materials, Department of Mechanical Engineering, Universiti Malaya, Kuala Lumpur 50603, Malaysia; h.metselaar@um.edu.my; 3 Petroleum Engineering Department, School of Chemical and Energy Engineering, Faculty of Engineering, Universiti Teknologi Malaysia, Johor Bahru 81310, Malaysia; akhmalsidek@utm.my; 4 Institute of Electronic Structure and Laser, Foundation for Research and Technology-Hellas, N. Plastira 100, Vasilika Vouton, GR-700 13 Heraklion, Crete, Greece

**Keywords:** unwanted wastes, nanomaterials, graphene, microwave, batteries, sensors, supercapacitors

## Abstract

It is no secret that graphene, a two-dimensional single-layered carbon atom crystal lattice, has drawn tremendous attention due to its distinct electronic, surface, mechanical, and optoelectronic properties. Graphene also has opened up new possibilities for future systems and devices due to its distinct structure and characteristics which has increased its demand in a variety of applications. However, scaling up graphene production is still a difficult, daunting, and challenging task. Although there is a vast body of literature reported on the synthesis of graphene through conventional and eco-friendly methods, viable processes for mass graphene production are still lacking. This review focuses on the variety of unwanted waste materials, such as biowastes, coal, and industrial wastes, for producing graphene and its potential derivatives. Among the synthetic routes, the main emphasis relies on microwave-assisted production of graphene derivatives. In addition, a detailed analysis of the characterization of graphene-based materials is presented. This paper also highlights the current advances and applications through the recycling of waste-derived graphene materials using microwave-assisted technology. In the end, it would alleviate the current challenges and forecast the specific direction of waste-derived graphene future prospects and developments.

## 1. Introduction

**Figure 1:**
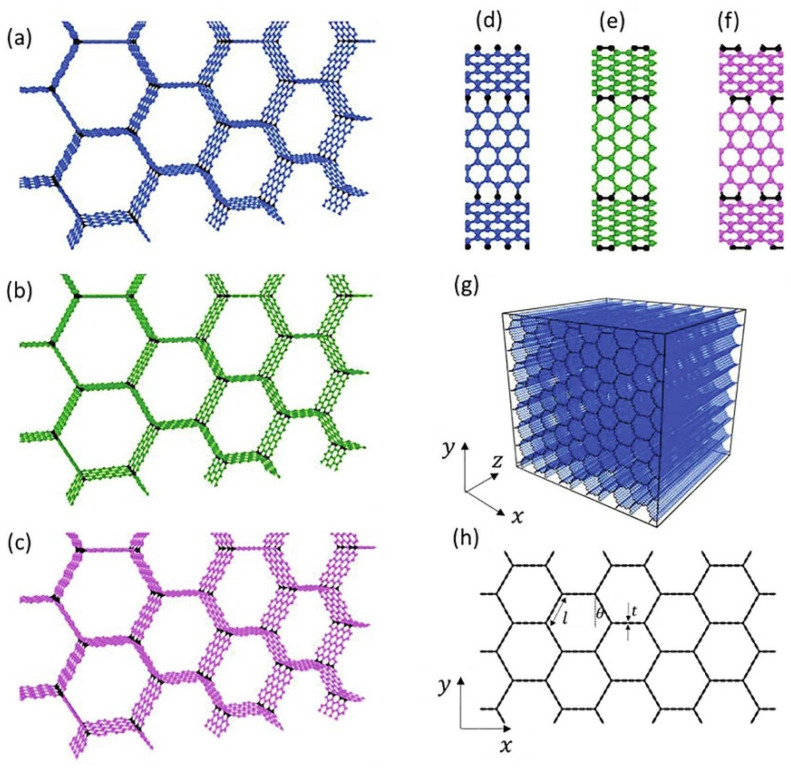
A variety of Graphene honeycomb 3D structures (**a**–**h**) [4].

Graphite has been utilized as the essential raw material in the production of graphene since its discovery. Graphite has an exceptionally anisotropic construction which leads to its in-plane and out-of-plane surface properties being very different [1]. Graphene is a layer of graphite. It is a solitary atom thick sheet of sp^2^ hybridized carbon atoms organized in a hexagonal grid structure with extraordinary properties, such as high surface area, high electrical conductivity, and excellent mechanical strength [2]. Due to its exceptional physical characteristics, such as its ultra-thin properties, significant nonlinearity, and electrical tunability, graphene is frequently used in combination with other materials to create tunable optical and other electronic devices [3]. Since each carbon particle has an unhybridized single bond, graphene has high native flexibility and electronic conductivity. Recently, 3D structures of graphene honeycombs have been studied through large-scale molecular dynamics simulations for mechanistic understanding and deformation behaviors as displayed in Figure 1 [4].

**Figure 2:**
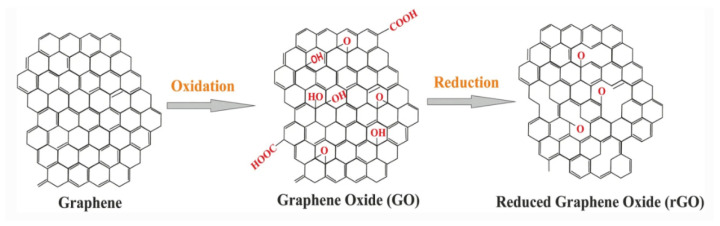
The structure of GO and rGO [6].

Graphene oxide (GO) is not a conductor. However, it can be reduced by heat processes into conductive reduced GO (rGO) [5]. rGO is conveyed by disposing of the oxygenated groups of GO, where GO is a variant of graphene adorned with functional groups [6]. Despite the fact that rGO is a derivative of graphene, the rigorous process of oxidation and reduction familiarizes harmed areas with the rGO sheets. There are unreacted functional groups attached to the rGO plane (Figure 2) [7].

Graphene, both single layer and multilayer, can now be manufactured in a variety of ways. The layers of graphene union are fabricated through a hierarchical or base methodology [8]. Graphite is composed of graphene layers. The graphene layers have two types of bond structures. The weak Van der Waal interactions hold the graphene bond layers together with a distance of around 0.341 nm between the adjoining graphene layers [9]. The Van der Waal interactions have a significant impact on the frequency of modes with relative movement between the layers in vibrational dispersion [10]. Some of the phenomena related to Van der Waal interactions include friction, surface tension, viscosity, adhesion, cohesion, and wetting [11]. The assembly of carbon atoms into a graphene arrangement is the bottom-up method of synthesis. The two methodologies have advantages and downsides that have been explored in literature [12,13]. In this study, we aim to review the available literature on the synthesis of graphene and graphene-based materials derived from wastes in the last decade. The focus of waste is biowaste, coal, and industrial waste as source materials. The specific synthesis method is microwave synthesis. Moreover, numerous characterization techniques have been discussed along with the emerging future prospects and recommendations.

## 2. Synthesis of Waste into Graphene Derivatives

### 2.1. Biowastes

It has become challenging in the 21st century to obtain clean, affordable, and reliable energy sources which are essential from both a financial and natural outlook. Biomass has been identified as one of the most favorable sustainable sources of energy [14]. Biomass is standard and normal material derived from plants and animals (microorganisms) and it contains stored energy from the sun [15]. Since plants and animals are classified as sustainable, the word “renewable” is applicable to both. Moreover, biomass is often time obtained from forestry, agricultural, industrial, household, and municipal solid wastes (MSW) [16,17]. Every year, various bio-waste from large-scale livestock or agricultural sources are dumped into the environment [18]. Biomass is mostly comprised of long chains of carbon, hydrogen, and oxygen compounds with a carbon fixation as high as 55% by weight [19]. The carbon content of biomass should be concentrated before it can be converted completely to graphene. The industry has utilized this strategy to make biochar. Biochemicals, biofuels, and even bio-vehicles are created from biomass utilizing heat treatment methods, such as gasification, carbonization, liquefaction, and pyrolysis [20]. Carbonization is a pyrolysis process that converts biomass into a carbonaceous, charcoal-like material [21]. On the other hand, graphitization is a method in which amorphous carbon is heated before being converted into three-layered graphite [22]. It ought to be noticed that the carbonization cycle habitually brings about amorphous carbon instead of graphite-like carbon. Pyrolyzed carbon exists in two forms which are hard and soft carbon. In fact, despite being heated to extremely high temperatures, hard carbon graphitization is yet to be achieved [23]. In the meantime, heat treatment readily converts soft carbon into graphite. In spite of the way that the properties of the converted carbon structures are similar to graphene, they are not unmodified graphene because of the presence of extra carbon components [24].

Thermal exfoliation and carbon growth are two methods for the thermal degradation of biomass [25]. The exfoliation technique with graphitized biomass incorporates breaking up the carbon structure by overcoming the Van der Waals forces, resulting in graphene sheets (GSs). This process is similar to the conversion of graphite into graphene with graphitized biomass substituted for graphite [26,27]. Table 1 shows examples of methods for the conversion of biomass into graphene derivatives.

### 2.2. Coal Waste

Coal is a unique carbon material that can be subdivided into lignite, bituminous coal, and anthracite [46]. Lignite and sub-bituminous coal are classified as inferior coal because of their high moisture content, high impurity, highly volatile matter substance, and low quantitative worth [47]. Coal is generally converted into fuel through various cycles, such as ignition, pyrolysis, gasification, and liquefaction [48]. The traditional methods have drawbacks which include a lack of energy efficiency and ecological contamination [49,50]. Subsequently, a high-esteem and earth-manageable technique in using coal is required [51]. Coal particles, in contrast with normal pieces of graphite and other precursors, contain a number of aromatic units as well as short aliphatic and ether bonds [52]. It is believed that coal might be a good option for creating carbon nanomaterials because of its staggered nanoarchitecture and explicit capabilities. Savitskii et al. [53] utilized anthracite coal and a thermo-oxidative technique to produce colloidal GO nanoparticle scatterings in size range from 122 nm to 190 nm. Pakhira et al. [54] showed that GO can be synthesized from low-grade coal. It was molded from the natural coalification of plant metabolites isolated by chemical exfoliation of cold HNO_3_. However, such GO sheets are bound to break into negligible round shapes of nanometers. It is striking that there is an expansion in the utilization of coal-derived nanomaterials for a variety of industrial applications [52].

Currently, the strategy for reprocessing coal into graphene is to initially convert huge molecule coal into an antecedent carbon source prior to synthesizing graphene. The precursor carbon source can be gaseous or a particular form [55]. Primer screening of crude coal, debasement expulsion, pyrolysis (dry refining), gasification, and liquefaction of coal steps in the preparation of a precursor carbon source [56]. Zhou et al. utilized a reactant graphitization-helped dielectric barrier discharge (DBD) plasma strategy to make Taixi anthracite-based synthetically inferred graphene as well as metallic nanoparticle-enhanced graphene sheets [57]. In this method, crude coal was graphitized at 2400 °C for 2 h (under Ar) directly with Fe_2_(SO_4_)_3_ as a catalyst followed by Hummers’ method oxidation into the corresponding graphite-like carbon oxides (TX-NC-GO and TX-C-GO, separately) [57,58].

### 2.3. Industrial Wastes

Malaysia is an emerging nation that relies on modern efficiency as one of its monetary donors. Different types of wastes are produced in industrial processes, including chemical effluents, industrial plants waste, paper waste, metals, concrete, sludge, electronic devices wastes, etc. [59]. A number of significant materials (e.g., graphite, Cu, Fe, and Zn) from industrial waste can be recuperated utilizing a hydrometallurgical technique called leaching [60]. The commercialization of graphite-based products has immensely improved during the twenty-first century [61]. It is due to their unique physical and manufactured properties, such as high chemical resistance, heat capacity, high electrical conductivity, and lubricity. These unique properties are suitable for various modern applications, such as contraptions, oils, and metallurgy [62].

A modified Hummers method was utilized to prepare to GO from graphite obtained from modern waste filtering [63]. Concentrated sulfuric acid (H_2_SO_4)_ and graphite (30 mL & 1 g) were mixed homogeneously in an ice bath for 30 min during the synthesis cycle. A total of 5 g of potassium permanganate (KMnO_4_) was added and mixed for another 15 min at temperatures below 10 °C [64]. The extent of KMnO_4_ was subsequently increased from 1:3 to 1:5 to speed up the oxidation rate. From that point onward, 8 mL of ultrapure water was added dropwise for 15 min, and the temperature of the mixture was kept under 98 °C for around 60 min. Finally, the oxidation reaction was obtained by adding 60 mL ultrapure water followed by 1 mL H_2_O_2_ [65].

## 3. Microwave Synthesis of Graphene Nanomaterials from Waste Materials

Microwave radiation is electromagnetic radiation with wavelengths ranging from 0.01 to 1 m and frequencies ranging from 300 MHz to 300 GHz [66]. Modern microwaves have two frequencies, 915 MHz and 2.45 GHz, while the consumed microwave only has one frequency, 2.45 GHz, and a wavelength of 12.25 cm [67]. Microwaves are widely used to heat materials that can absorb and convert microwave radiation to heat [68]. These dipolar particles that are changed can quickly rearrange toward the electric field, leading to expanded inward atomic contact, and volumetric warming of the whole substance [69]. As a result, microwave-assisted technology is able to provide a quick and efficient method of evenly heating the material or system from within. The conventional heating system, on the other hand, is relatively slow and ineffective [70].

**Figure 3:**
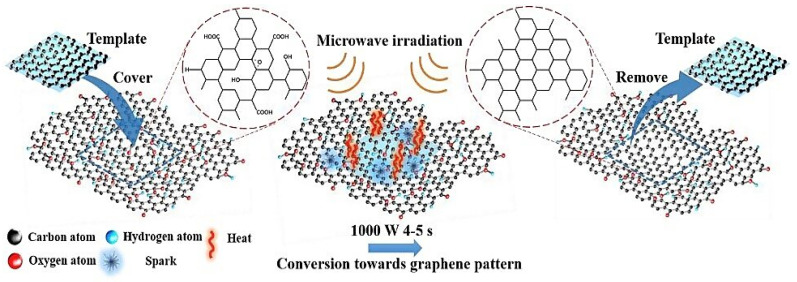
Schematic illustration of the synthesis of graphene and graphene-based composites with the assistance of microwave irradiation [77].

Graphite or GO, is a typical wellspring of GSs, which are made from a conventional or modified Hummer’s method [71]. Hummers’ method is the most widely used method in the synthesis of GO through a mixture of concentrated H_2_SO_4_ and KMnO_4_ [72]. Since then, numerous modified versions have been developed. However, the experimental procedures are mainly very similar to the original Hummers method. Oxidation is achieved using KMnO_4_ and the reaction is stabilized by adding hydrogen peroxide into the solution [73]. A few hazardous reducing agents, such as hydrazine (N_2_H_4_) and NaBH_4_, are normally utilized in substance methodology to reduce GO. Thermal treatment, on the other hand, does not require the utilization of hazardous reducing agents making it a more attractive option [74]. The microwave-assisted technique has acquired ubiquity as an alternative to conventional graphene preparation. It treats GO or normal graphite in a microwave or microwave plasma-assisted chemical vapor deposition (MPCVD) framework which utilizes microwave-assisted solvothermal/aqueous strategies [75]. Microwave radiation provides a quick and uniform heating rate that leads to fast particle nucleation and growth which may reduce the reaction time that eventually led to significant energy saving [76]. Figure 3 portrays one potential microwave-assisted strategy for graphene synthesis. Microwave illumination produces very high temperatures and tensions, and energy is transferred directly into the GO [77]. Furthermore, the interaction of polar solvents with the surface oxides on GO sheets is the key factor in determining deposit regularity [77].

Furthermore, the reduction degree of GSs was further enhanced, and the functional groups on the surface of GO are successfully lowered [78]. There are several obvious advantages to producing graphene using microwave technology. Firstly, the advantage of microwave-assisted heating over traditional heating methods is its uniform and rapid heating of the reaction mixture [79]. In addition, microwave-assisted heating can significantly improve the transfer of energy directly to the reactants, resulting in an instantaneous internal temperature rise [80]. Furthermore, microwave technology enables the use of environmentally friendly solvents, resulting in cleaner products that do not require additional purification steps [81]. Since it involves a quick warming and very fast rate of crystallization to create the ideal nanocrystalline items, microwave illumination has recently been proposed as a valuable procedure for delivering carbon-related composites with uniform scattering as well as size and morphology control [82]. Table 2 shows examples of waste materials in graphene derivatives by using the microwave method.

## 4. Characterization Techniques

### 4.1. X-ray Diffraction (XRD) and X-ray Photoelectron Spectroscopy (XPS)

**Figure 4:**
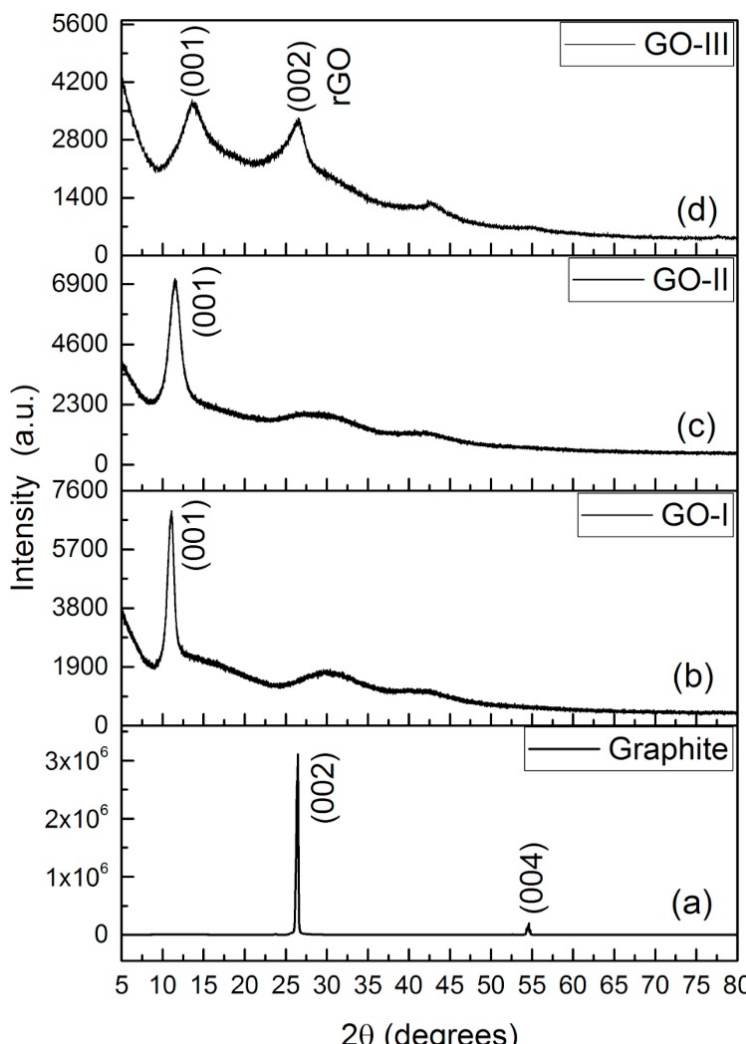
XRD patterns of graphite (**a**), GO-I (**b**), GO-II (**c**) and rGO (**d**) [109].

Firstly, XRD is a reliable technique for the structural analysis of GO. This analysis can be used to assess the pattern/shape and crystallinity of GO [103]. XRD also is comparable to a fingerprint that is unique for each sample or species. This is due to the evaluation of achieved data which can be compared with the database results to identify that material [104]. In spite of the fact that XRD is certainly not an optimal device for recognizing single-layer graphene, it can be used to recognize graphite and graphene tests. In the XRD design, the unblemished graphite has a basal reflection (002) peak at 2θ = 26.6° (*d* spacing = 0.335 nm). Later, the oxidation of graphite into graphite oxide shows middle basal (002) reflection peak moves to 11.2°, corresponding to a d spacing of 0.79 nm [105]. The increase in interlayer space is due to water atoms intercalating between the oxidized graphene layers. The presence of metallic mixtures in graphene structures was analyzed utilizing XRD examination. In addition, an x-beam connection with a graphitic translucent stage produces a diffraction design [106]. Non-covalent functionalization of rGO with two poly ionic fluids (PIL), poly (1-vinylimidazole) (PVI), and 2-bromopropionyl bromide resulted in the disappearance of a sharp GO diffraction peak at 2θ = 11.8° in PIL-rGO diffractograms [107]. This trend is predictable with the detailed information and a slight expansion in the power of the GO trademark top in 2θ = 44.5° (101) which relates to the basal reflexing plane of the tri-layered graphite [108]. Figure 4 displays the XRD profiles of graphite, GO-I, GO-II, and rGO. The formation of GO was confirmed by the diffraction peak at 2θ = 11.01° at a reflection plane (001). A diffraction peak that appeared at 2θ = 26.8° at a reflection plane (002) after the thermochemical treatment confirmed the reduction of GO [109]. This diffractogram demonstrated the disappearance of the GO peak, providing evidence that GO was converted into rGO. In addition, GO was prepared using different ratios of acids (I and II) as shown in Figure 4 [109,110].

**Figure 5:**
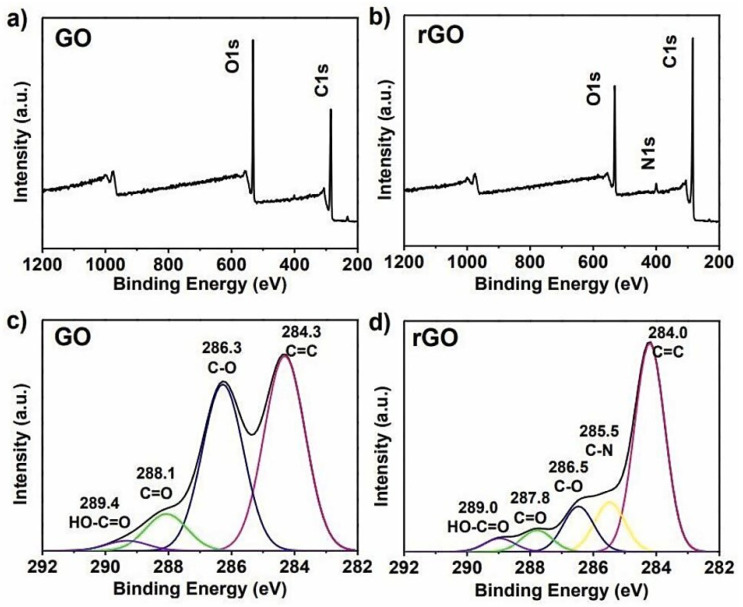
XPS spectra of (**a**) GO and (**b**) rGO. C1s XPS spectra of (**c**) GO and (**d**) rGO [117].

XPS is one of the most common techniques used to study the relative amount of carbon, oxygen, and functional groups present in GO and electrochemically rGO (ErGO). It is an accurate technique to determine the amount of carbon and oxygen compared to elemental analysis because it is difficult to fully dehydrate a GO sample [111]. This is a quantitative and reliable technique in removing electrons from the C 1s and O 1s levels of graphene using X-rays and the energies of the emitted electrons are determined by the atomic composition of the material [112]. XPS can quantify the different types of carbon functionalities present and indicate the formation of chemical bonds, and evaluate the physisorption of molecules through the O/C ratio [113]. This quantification is critical to correlate the graphene-based materials’ chemical properties versus their performance, for example, in permeability [114], water purification [115], or bio-sensing [116]. Furthermore, the surface chemistry and binding sites of both electrically conducting and non-conducting materials are also studied by XPS. It is possible to characterize the networks and bonds in the material sample. The photoelectric effect serves as the basis for the theory. Additionally, XPS can shed light on the atomic composition’s percentage. Figure 5 displays the GO and rGO of the XPS spectra that exhibit distinctive patterns which reveal their chemical composition [117].

The C(1 s) and (O1 s) peaks, which are located at about 285 and 532 eV, respectively, in the XPS full scan spectra of GO and rGO, are discernible [118]. The bonding involved is further highlighted by the deconvolution of the core orbitals of C(1 s) and O(1 s) [119]. Peaks for C=O, O=C-OH, C=C and C-C bonds are respectively visible in the C(1 s) deconvolution for GO at binding energies of 287, 289, 284, and 285 eV. The C-OH and C-O-C groups have peaks on the O(1 s) deconvolution curve for GO at 532 and 533 eV, respectively [120].

Table 3 has highlighted the GO and rGO binding energy values. In the case of rGO, these peaks show up with low intensity, confirming the reduction of GO. As a result, the peaks in rGO become narrower when GO is reduced to rGO. Additionally, it appears intense to restore the π-conjugation in the rGO peak at 284 eV which corresponds to C=C. The deconvoluted peaks in rGO shift to a binding energy value greater than GO for O(1 s).

The appearance of distinctive peaks in XPS can be used to verify that graphene has been successfully non-covalently functionalized [121]. According to Khan et al., two distinct peaks at 729 and 715.3 eV can be used to identify the presence of magnetic nanoparticles anchored on the GO surface [122]. Furthermore, it is noted that the XPS C1 spectrum after Fe3O4-functionalization shows peaks associated with C=O (285 eV), C=C (286.2 eV), and C-O-O (289 eV) bonds. XPS can also be used to identify active sites and further illuminate associated reaction mechanisms in graphene-based catalytic materials. This is best illustrated by the direct observation of active sites during the oxygen reduction reaction (ORR) over nitrogen-doped graphene (NG) catalysts [123]. Even though many simulation results showed various reaction pathways and adsorption sites for ORR over NG, the actual mechanism is still in dispute, primarily because there is not any direct evidence of the detection of intermediate species or active sites [124].

### 4.2. Other Characterization Methods

#### 4.2.1. Raman Spectroscopy and Fourier-Transform Infrared Spectroscopy (FTIR)

**Figure 6:**
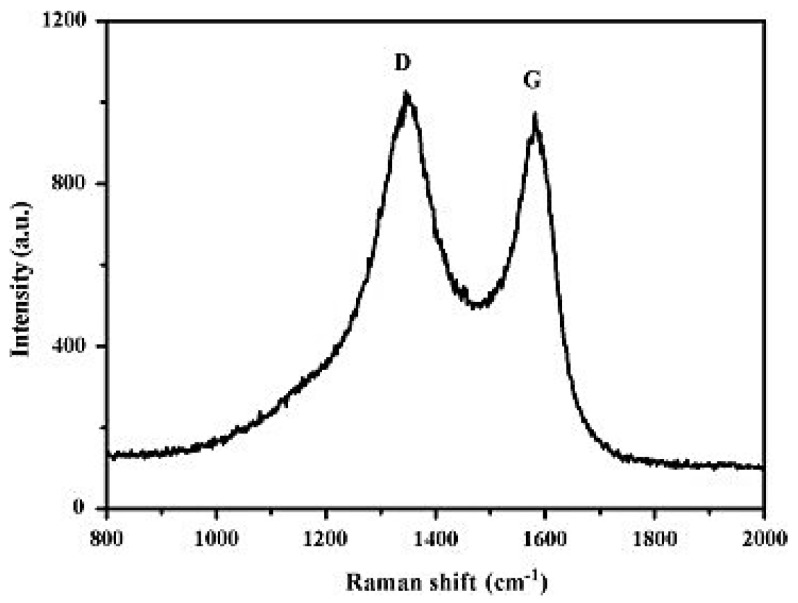
Raman spectra of samples at various stages of processing [132].

Raman spectroscopy detects the transformation in energy connected with the Stokes and anti-Stokes transitions between the scattered photons. It is a non-destructive technique that provides information on chemical structure and molecular interactions by the combination of light within the bond of material [125]. Moreover, Raman spectroscopy is one of the most useful assets for concentrating on the construction and nature of carbon-based materials, for example, graphene [126]. It is a powerful, quick, delicate, and logical technique for giving subjective and quantitative information to graphene-based materials [127]. Raman spectroscopy is a significant instrument for deciding the quantity of graphite layers and the level of graphitization [128]. Graphene shows D, G, and 2D bands for the most parts in Raman analysis [129]. The D band is commonly situated around 1350 cm^−1^ and addresses the level of defects in the graphite. The higher the D band, the more defects in the graphite are observed [130]. The G band is linked to the in-plane vibration of sp^2^ hybridization of carbon atoms which is located near 1580 cm^−1^. The 2D peak, also known as G’, represents the number of graphene layers and is observed at 2700 cm^−1^ [131]. Figure 6 depicts the Raman spectra of graphene reduced with various reduction conditions which reflect the significant structural changes that occur during each stage of the electro and thermal processing [132].

Pristine graphene is a carbon allotrope, and no signal can be collected using FTIR. Graphite oxide exfoliation is one of the primary routes for preparing practical graphene which supports catalytic research, and the oxidation step is critical [133]. As a result, many functional groups may remain in graphene-based catalysts even after being “completely removed”, having a significant impact on catalytic performance [134]. Therefore, it is important to evaluate the reduction level. FTIR is one of the most efficient and simple methods for investigating residual functional groups [135]. Other than that, the method to determine the bonding configuration of different types of oxygen is FTIR analysis. Additionally, FTIR is a tool that complements Raman spectroscopy. The identifiable functional groups do not show any distinctive peaks in the pristine graphite FTIR spectrum [136]. It only shows two peaks at about 1610 and 450 cm^−1^ which are attributed to the vibration of adsorbed water molecules (the O-H stretching) and the skeletal vibrations from graphite domains, respectively (the sp^2^ aromatic C=C) [137]. The oxygenated GSs may exhibit a variety of absorption bands or characteristic peaks ranging from 900 to 3500 cm1 following treatment with oxidizing agents [138]. These include the stretching vibrations of epoxy C-O groups (1000–1280 cm^−1^), alkoxy stretching vibrations (1040–1170 cm^−1^), O-H stretching vibrations (3300–3500 cm^−1^), O-H deformation peaks (1300–1400 cm^−1^), and carboxyl peaks (1700–1750 cm^−1^) [139]. Notably, between 1600 and 1650 cm^−1^, the aromatic C=C peak was visible. This peak is a result of the sp^2^ domains in the unoxidized region of the graphite, and the vibration that is produced there is known as skeletal vibration [140].

#### 4.2.2. Atomic Force Microscopy (AFM)

**Figure 7:**
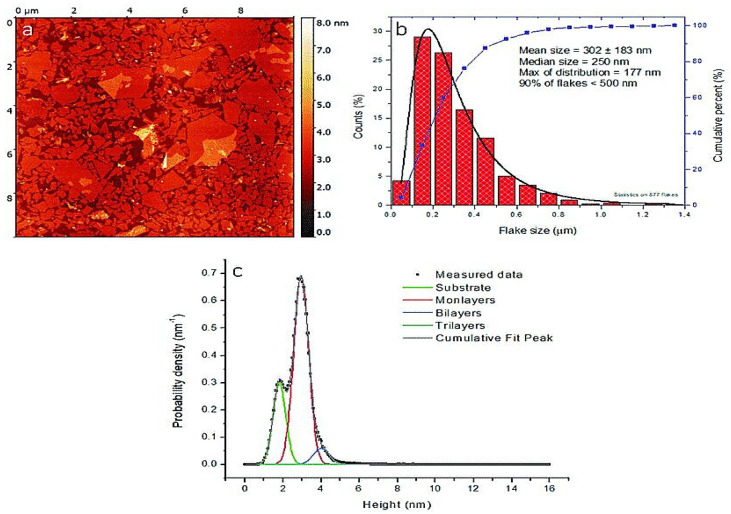
Purified GO: (**a**) atomic force microscopy (AFM) image, (**b**) GO size distribution, and (**c**) AFM scan height analysis [150].

As a result of the limits of scanning tunneling microscopy (STM), such as the requirement for conductive examples, atomic force microscopy (AFM) was created in 1985 [141]. AFM is a multifunctional instrument that can envision the topography of a sample, measure its roughness, and distinguish the various periods of a composite [142]. It is widely used to measure the adhesive strength and mechanical properties of materials. It requires the utilization of conductive tips that act as top terminals as well as related to programming. Furthermore, nanoindentation can be utilized to quantify mechanical properties, such as Young’s modulus and hardness [143]. AFM is broadly utilized in materials science [144], life science, and other disciplines [145]. As AFM innovation progresses, perception goal improves, and application scope extends and also more quantitative investigation of noticed pictures has started [146]. For instance, in the field of biomedicine, most exploratory examinations have zeroed in on the connection between the design and related elements of natural macromolecules, especially nucleic acids and proteins [147]. AFM in materials science can provide data related to the three-layered morphology and surface roughness of a material surface, as well as the distinction in the distribution of actual properties on the material surface, for example, morphological analysis [148] and dielectric constant [149]. A modified Langmuir–Schaefer deposition method was used to create a thin monolayer film suitable for imaging in the samples for AFM measurements. Figure 7 shows a representative AFM image of the GO monolayer deposited on the Si substrate as well as the corresponding size distribution of the GO sheets [150].

#### 4.2.3. Scanning Electron Microscopy-Energy Dispersive X-ray Spectroscopy (SEM-EDS)

**Figure 8:**
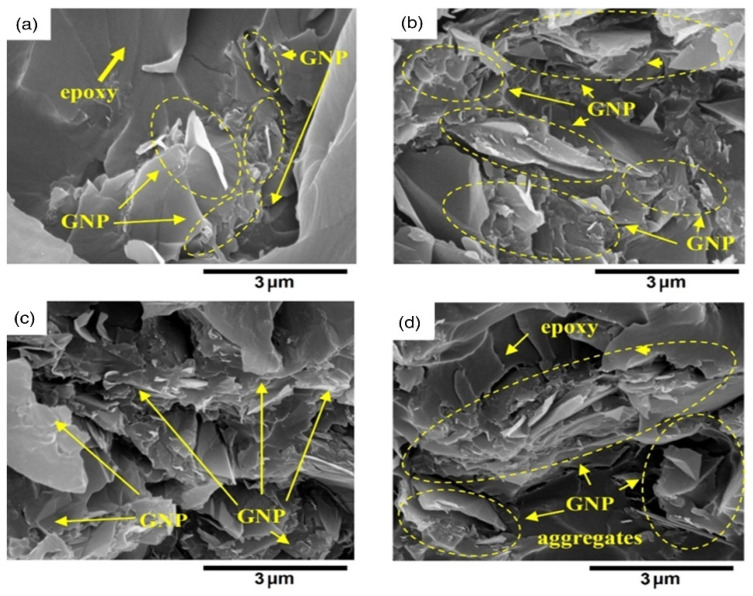
SEM images of GNP fracture surfaces in epoxy at loadings of (**a**) fGNP = 1%, (**b**) fGNP = 2%, (**c**) fGNP = 10%, and (**d**) fGNP = 20% [158].

Since it can rapidly examine/imagine the morphology of a huge sample, electron microscopy is broadly utilized in everyday schedule examinations [151]. A potential difference accelerates thermionic electrons transmitted by a tungsten fiber (cathode) close to the anode (1.0–30.0 kV). A condenser and objective electromagnetic focal points are utilized to adjust the bar to the example under vacuum (105 Dad) [152]. Secondary and backscattered electrons are transmitted during the output, as well as Auger electrons and X-rays, and their interaction with electrons which changes them completely to grayscale pictures. Pictures of the sample are given by secondary and backscattered electron identifiers, while compositional data is given by the X-ray spectrometer [153]. Secondary electrons are fundamentally created by the outer shell’s inelastic scattering, while backscattered electrons are delivered by the primary electrons [154]. To avoid surface and underlying damage from the rays, delicate examples, such as polymers, need to be treated carefully. Nonconductive examples require surface pre-treatment and the sample is normally covered with a gold or carbon overlayer [155]. Due to the oxygenated epoxy groups of GO, it shows multilayers with some wrinkles [156]. SEM images provide 3D visualization of nanoparticles morphology, dispersion in cells, and other matrices. Lateral dimension and rapid analysis of nanoparticles element composition and surface flaws, such as cracks, etching residues, differential swelling, and holes can also be seen [157]. Figure 8 shows SEM images of protruded GNP produced by GNP debonding from the polymer matrix upon failure as indicated by circles when GNP loading is increased to 10% and 20%, respectively. It has been observed that while GNP loading is increased to 10% and 20% (Figure 8c,d), the fractured surfaces become much coarser [158].

#### 4.2.4. Transmission Electron Microscopy (TEM) and High-Resolution Transmission Electron Microscopy (HRTEM)

**Figure 9:**
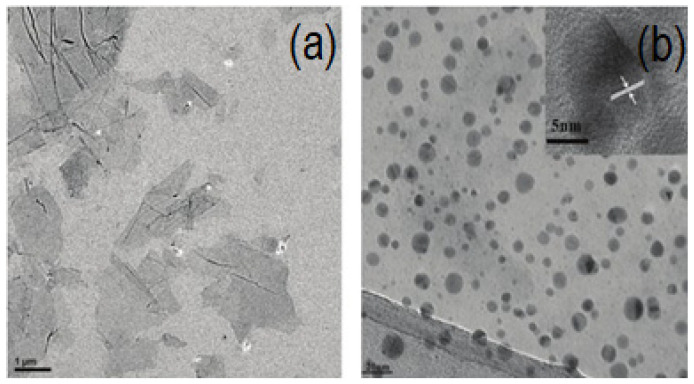
TEM image of bare GO (**a**) TEM and HRTEM image of rGO-Au (**b**) [165].

TEM is best known for imaging a specimen’s morphology, a wide variety of other combined techniques are also available in TEM to extract chemical, electrical, and structural data. For instance, local diffraction patterns can be measured using the parallel electron beam of the TEM which can offer precise measurements of the crystal system and parameters [159]. Furthermore, the transparent, corrugated, or wrinkled structure of the two-dimensional (2D) GO and rGO nanosheets is visible under the TEM [160]. It is also described as having the morphology of an ultrathin silk veil with folds and scrolls along its edges and it is attributed to graphene’s inherent properties [161]. A highly effective method for characterizing the structure of graphene is HRTEM. It is a special tool for describing graphene’s atomic structures and interfaces. It has been used to observe graphene flakes in a fraction of a micron and to reveal the fine chemical structure of GO [162]. Based on a TEM image of the folds formed at the edge, HRTEM also provides data on the number of graphene layers. Graphene’s electron diffraction pattern can also be used by HRTEM to identify its crystalline nature [163]. It is noteworthy that HRTEM can reveal the quantity of layers present in various areas of the sheets [164]. The measured lattice spacing of single-layer graphene using this method is 0.236 nm [165]. Figure 9 shows TEM and HRTEM images of rGO.

#### 4.2.5. Field Emission Scanning Electron Microscopy (FESEM)

**Figure 10:**
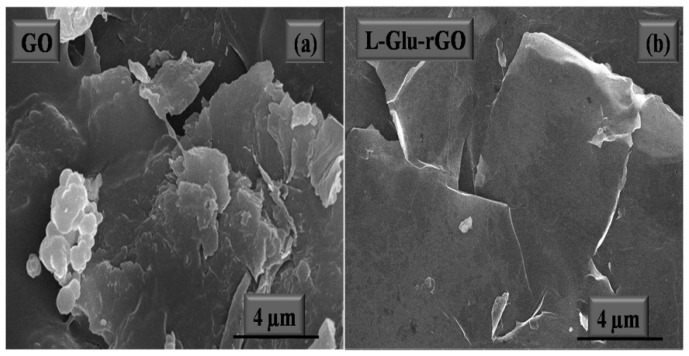
FESM micrograph of GO (**a**) and rGO (**b**) [168].

The image of the materials’ microstructure is captured using the cutting-edge technology known as FESEM. Gas molecules have a tendency to disturb the electron beam and the emitted secondary and backscattered electrons used for imaging and FESEM is typically carried out in a high vacuum [166]. The difference between the surface morphology of GO and rGO was further demonstrated by FESEM analysis [167]. It has been demonstrated that the rGO’s FESEM image from Figure 10 has more wrinkles than GO [168]. The removal of oxygenated functional groups from the GO surface during the reduction process was supposed to be the cause of the corrugations on the rGO surface [169].

## 5. Future Prospects

**Figure 11:**
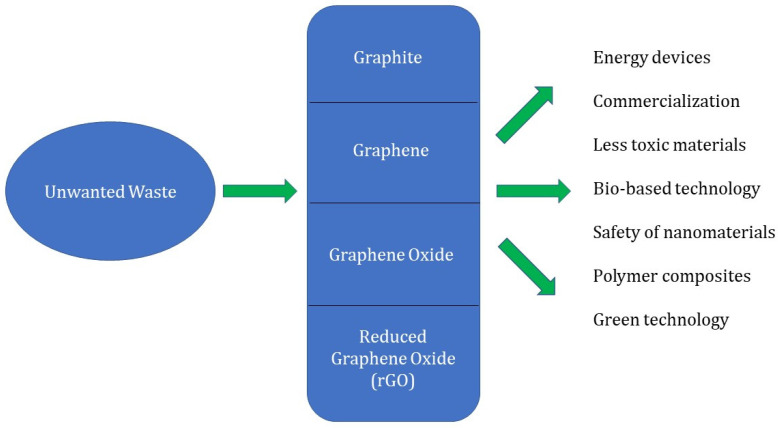
Future prospects in graphene synthesis from a variety of wastes.

Even though scientific interest in graphene has increased for a variety of applications, there are still several significant obstacles and challenges that need to be addressed and overcome. One of the critical issues is the reproducibility of waste materials into graphenaceous materials. Improved morphological properties should be combined with procedures that are both scalable and affordable. Excitedly, there is a sustained interest in the synthesis of materials based on graphene and the evaluation of their production and fusion with other materials. Although waste precursors have been the subject of recent studies, none of them have yet been able to be marked into commercially available products. Figure 11 shows the future prospects in graphene synthesis from a variety of wastes. Noteworthy future prospects include;

- Optimization of process variables and techniques to regulate the size, quality, and morphology of graphene-derived materials from waste materials.
- Improved synthetic concepts and methods are highly inspiring and necessitate commercial research involving renewable and biodegradable waste materials.
- Well-ordered oxidation/decrease and functionalization are expected for calibrating material properties, for example, band hole, electrical conductivity, and mechanical properties [170].
- Controlled graphite, GO, and rGO adjustment is in this way basic for widening the utilizations of graphene-based materials.
- To survey the wellbeing risk related with graphene and its subsidiaries, the poisonousness and biocompatibility of these unique carbon structures and their subordinates should be examined [171].
- Due to its extensive property, graphene preparation is a crucial area for material scientists. As a result, the scientific community should focus on advanced and novel microwave instruments which would be a great substitute of toxic and harsh chemicals
- To explore more variations that involving novel synthetic techniques, high purity GO for its mass production.
- There should be more consideration to lessen the cost effects of graphene derivatives.
- There should be more emphasis on the high yield and purity of graphene derivatives using a variety of wastes through microwave synthesis.
- This may also lead towards the excellence of functionalization, such as ID, 2D, and 3D graphene members, to fabricate waste materials into graphene-based structures with enhanced functionalities and high surface areas [172].
- Improving synthetic ideas and microwave approaches are remarkably motivating and requires further investigations by recycling waste materials for the optimization of parameters, such as time, power, and frequency.
- Further analysis of microwave synthesis and applications should be explored where the waste-based graphene derivatives can be utilized and, thus, the structures and properties can be modified as per the industrial demands.

## 6. Conclusions

Due to graphene’s industrial significance, there is great concern about its sources and synthesis methods. These variables affect the price of graphene, and its industrial applications are constrained. The current study presents an overview of various types of wastes followed for the synthesis of graphene-based materials. Graphene creates wonders with its intriguing qualities and great attention from researchers all over the world. GO isolation has been established for more than ten years. However, the process is a continual exploration of variations involving novel synthesis techniques, and highly pure GO for its mass production and commercialization. The major attention is to have a process that is cost effective and economical. The literature is rich with important process parameters, their optimizations, and the synthesis of GO from a variety of waste which is useful for a wide range of applications. We have narrated literature that lists the synthetic routes for GO, particularly microwave synthesis, as well as different characterization methods. Although waste biomass-inferred graphene is one more encouraging material with various applications, its synthesis method is still open to be explored. Accordingly, more examination is expected to exhibit the best strategy for creating graphene with the best properties and optimization. In addition, successful and cost-effective planning may lead to the use of graphene in a wide range of applications from energy to the environment. Furthermore, material progress always demonstrates a superior effect in any field. Due to its diverse properties, graphene preparation is an important area for material scientists. As a result, the scientific community will always give attention to effective and efficient graphene preparation. The improvement of graphene through green combination addresses a huge progression in graphene innovation. The cost of producing graphene in large quantities could be reduced in alternative ways using carbonaceous wastes as raw materials. The production of graphene for industrial applications should successfully utilize a variety of environmentally hazardous solid waste precursors. Since waste-derived graphene might have impurities, additional purification procedures are needed. Future research is therefore required to increase graphene production with better yield and properties.

## Figures and Tables

**Table 1:** Methods for the conversion of biomass into graphene derivatives.

Waste Sources	Methods	Temperature	Atmosphere	Time	Graphene Derivatives	References
Petals of lotus and hibiscus flowers	Chemical vapor deposition (CVD)	800–1600 °C	Air	0.5 h	graphene	[28]
Newspaper	Carbonization	450 °C	Air	5 days	graphene	[29]
Chitosan	Pyrolysis, Chemical activation	800 °C 900 °C	N_2_ gas	3 h 2 h	graphene	[30]
Camphor leaves	Pyrolysis	1200 °C	Nitrogen gas	4 min	graphene	[31]
Wheat straw	Hydrothermal, Pyrolysis, Pyrolysis	150 °C 800 °C 2600 °C	Air N_2_ gas Ar gas	6 h 3 h	graphene	[26]
Oil palm leaves & Palm kernel shell	Pyrolysis	700 °C	N_2_ gas	3 h	GO	[32]
Oil palm fiber	CVD & Pyrolysis	1020 °C	Ar and H_2_ gas	30 min	graphene	[33]
Rice husks	Chemical activation	400 °C 800 °C	Air	2 h	graphene	[34]
Palm oil	Pyrolysis	900 °C	Ar gas	10 min	GO	[35]
Spruce bark	Hydrothermal Pyrolysis	180 °C 1000 °C	Air N_2_ gas	12 h 2 h	graphene	[36]
Mango peel	Pyrolysis	750 °C	H_2_ gas Ar gas	15 min	graphene	[37]
Macademia nut shell	Hydrothermal Pyrolysis	180 °C 800 °C	Air Argon gas	12 h 2 h	graphene	[38]
Soybeans	Pyrolysis	800 °C	Nitrogen gas	2 h	graphene	[39]
Empty fruit brunch	Pyrolysis Graphitization	350 °C 900 °C	N_2_ gas	2 h	graphene	[40]
Bengal gram bean husk	Pyrolysis	400 °C 850 °C	Nitrogen gas	2 h	graphene	[41]
Populus wood	Pyrolysis	950 °C	Nitrogen gas	1 h	graphene	[42]
Lignin biomass	Hydrothermal	180 °C	Air	12 h	graphene	[43]
Walnut shell	Pyrolysis	700 °C	Argon gas	4 h	graphene	[44]
Coconut shells, Oil palm empty fruit bunches (OPEFB), Rice husks	Carbonization	250, 300, 350, 400, 450 °C 105 °C 250, 300, 350 °C	NaOH NaOH Air	2 h 24 h 2.5 h	GO	[45]

**Table 2:** Examples of waste materials into graphene derivatives by using microwave method.

Types of Waste	Microwave Experimental Parameters				Characterizations	Applications	References
	Power	Time	Reagents	Frequency			
Graphite powder	700 W	60 s			XPS, XRD and TEM	Fuel cell catalysts	[83]
Cellulose	950 W	2 h	H_2_SO_4_	-	XRD	Biobased GO Quantum dots (GOQD)	[84]
Sugarcane bagasse (dried) & bulk	700 W 800 W	2 min 10 min	H_2_SO_4_ Argon gas	2450 MHz	FESEM, XRD, XPS and Raman spectroscopy SEM, Raman spectroscopy	Li-ion battery (LIB)	[85] [86]
Betalain from dragon fruit	100 W	10 min		-	ANOVA and BBD design matrix	Coloring food product	[87]
Spent tea waste	100-900W	15-180 min		-	TEM, XPS and FTIR	Graphene quantum dots (GQDs)	[88]
Waste palm	700 W	5 min		-	FESEM, XRD, XPS, TEM and Raman spectroscopy	Supercapacitor	[89]
Poly (Ethylene terephthalate)	700 W	300 s	Iron nano-particles	2450 MHz	XPS, Raman spectroscopy, FESEM, SEM, HRTEM and EDX	Bisphenol-A removal from contaminated water	[90]
Coconut shells	800 W	10, 20, 30, 40 min	L-ascorbic acid	2.45 MHz	FTIR, SEM, EDAX, XRD, LCR-Meters.	Effects of microwave irradiation	[91]
Sorghum stalk	700 W	3 min		-	SEM, XRD, XPS, TEM and EDS	Supercapacitors	[92]
Coconut coir and coconut shell		12 h		-	XRD, XPS, TEM and SEM	Electrical transportation system	[93]
Bovine blood waste	700 W	10 min		-	XPS and Raman spectroscopy	Food industry	[94]
Coconut shells	900 W	15 min		-	XRD and Raman spectroscopy	A hybrid gas sensor from room temperature	[95]
Waste PET bottle	600 W	2 min		-	EDX, FTIR XRD and SEM.	Tetracyclines removal	[96]
Disposable mask				-	SEM microscopy	Composite materials	[97]
Grass waste		8 h		-	FTIR, TEM, Raman, AFM, XPS, UV-Vis and HRTEM	Nonlinear optical applications	[98]
Styrofoam waste	1100 W	30 min		2.45GHz	TEM, Raman, XRD, FTIR and SAED	Nonpolar GQDs-based hydrophobic coating	[99]
Bamboo waste	2000 W	25 min	K_2_CO_3_	-	XRD, TEM, SEM and XPS	Biochar containing graphene (BCG)	[100]
Melamine sponge and arjuna bark	700 W	10 min		-	FTIR, XPS and TEM	Cell imaging and H_2_O_2_ sensing	[101]
Toner powder waste	350 W	30s		-	Raman, FTIR, UV-Vis spectrometer and FETEM	Color converting film	[102]

**Table 3:** Binding energy values of GO and rGO in (eV) from the XPS plot [117,119].

Bond	GO		rGO	
	C (1 s)	O (1 s)	C (1 s)	O (1 s)
C=C	284	-	Increase in intensity	-
C=O	287	-	Decrease in intensity	-
C-O-H	285	532	Decrease in intensity	Narrowing of peaks
C-O-C	-	533	-	Decrease in intensity

## Data Availability

Not applicable.

## Abbreviations

GO	Graphene oxide
rGO	Reduced graphene oxide
MSW	Metropolitan strong squanders
GSs	Graphene sheets
MPCVD	Microwave plasma assisted chemical vapor deposition
PIL-rGO	Poly ionic liquid reduced graphene oxide
ErGO	Electrochemically reduced graphene oxide
DBD	Dielectric barrier discharge
TX-NC-GO and TX-C-GO	Coal-based graphite-like carbons oxides
XRD	X-ray diffraction
XPS	X-ray photoelectron spectroscopy
TEM	Transmission electron microscopy
FESEM	Field emission scanning electron microscopy
ANOVA	Analysis of variance
BBD Design	Box-behnken design
SEMEDAX	Scanning electron microscopy energy dispersive X-Ray
EDS	Energy dispersive spectroscopy
SEM	Scanning electron microscopy
FTIR	Fourier transform infrared spectroscopy
AFM	Atomic force microscopy
SAED	Selected area (electron) diffraction
FETEM	Field-emission transmission electron microscope
